# Validation of the Tunisian Empathy Scale for Children (TESC) in General Population and Children With Autism Spectrum Disorder

**DOI:** 10.3389/fpsyt.2022.903966

**Published:** 2022-07-18

**Authors:** Hela Ben Youssef, Soumeyya Halayem, Malek Ghazzai, Selima Jelili, Hager Ben Mansour, Olfa Rajhi, Amal Taamallah, Selima Ennaifer, Malek Hajri, Zeineb Salma Abbes, Radhouane Fakhfakh, Ahmed Nabli, Asma Bouden

**Affiliations:** ^1^Department of Child and Adolescent Psychiatry, Razi Hospital, Manouba, Tunisia; ^2^Faculty of Medicine of Tunis, University of Tunis El Manar, Tunis, Tunisia; ^3^Service Universitaire de Psychiatrie de l'Enfant et de l'Adolescent du Centre Hospitalier de Versailles, Paris, France; ^4^Department of Preventive Medicine, Abderrahmen Mami Hospital, Ariana, Tunisia; ^5^ReMotion Research and Development, Lille, France

**Keywords:** empathy, child, validation study, autism (ASD), questionnaire

## Abstract

**Background:**

Several empathy assessment tests have been proposed worldwide but none of them took into account cultural variations that seem to affect empathic manifestations. The aim of this study was to create and validate an empathy assessment questionnaire for school-aged Tunisian children entitled “Tunisian Empathy Scale for Children” (TESC).

**Methods:**

An evaluative cross-sectional study was conducted. The questionnaire was administered to parents of 197 neuro-typical children and 31 children with autism without associated intellectual deficits, aged between 7 and 12 years. Validation steps included: face validity, content validity, construct validity, and reliability study. A ROC curve analysis was used to investigate the diagnostic performance of the TESC.

**Results:**

Face validity was verified with an expert panel. Content validity was examined, and 11 items were removed as irrelevant or not assessable by parents. Exploratory factor analysis extracted four domains that explained 43% of the total variance. All these domains were significantly correlated with the total score (*p* < 10^−3^) and are, respectively: empathic behaviors, affective empathy, cognitive empathy, and a combined affective and cognitive domain. The reliability study showed a satisfactory level of internal consistency of the TESC, with a Cronbach's alpha of 0.615.

The diagnostic performance of the TESC in relation to autism was evaluated by the ROC curve with a sensitivity and specificity of 84.3 and 62.1%, respectively, for a total score of 16.

**Conclusion:**

A 15-item questionnaire assessing empathy in a multidimensional and culturally adapted way was obtained. The psychometric qualities of the TESC were satisfactory.

## Introduction

Social cognition refers to the set of skills and cognitive processes that regulate interpersonal relationships. It is a composite construct, which includes several interdependent dimensions among which empathy occupies a central place (1). Empathy is often defined as the ability to infer mental and emotional states of others and to respond appropriately and effectively (2). Even though there is no consensus on its definition, many authors define it in a multidimensional way by attributing to it at least three main components: affective, cognitive, and behavioral (3). Empathy is impaired in several neurological and psychiatric entities such as frontotemporal lobar degeneration, Alzheimer's disease, Parkinson's disease, Huntington's disease, schizophrenia, and autism spectrum disorder (ASD). Each of these pathologies seems to have a particular empathy profile. This hypothesis is supported by advances in functional neuroimaging which indicate different brain activation zones according to the pathology concerned in response to a given stimulus (4, 5). Hence, several researchers have developed empathy assessment scales that could be commonly used like the Empathy Quotient (6), Empathy assessment index (7), The cognitive, affective, and somatic empathy scales (CASES) for children (8); The children's empathy quotient and systemizing quotient (9), Griffith Empathy Measure (10), empathy components questionnaire (11). These scales have conformed over the years to the multidimensional conception of empathy. However, these scales do not consider cultural differences that seem to influence empathic manifestations (12). The goal of this study was to create an empathy assessment tool adapted to the Tunisian context entitled “Tunisian Empathy Scale for Children” (TESC), and to validate it on a general pediatric population and on a clinical population (children with ASD). This work falls within the Tunisian battery of social cognition assessment tools which already includes two validated tests: “Tunisian social situation instrument” (13) and “Tunisian test for facial emotions recognition” (14) designed as downloadable applications on Android.

## Methods

### Participants

An evaluative cross-sectional study was conducted in a general and a clinical population of children with ASD without intellectual and language impairment according to DSM-5 (15). Children of both groups were aged between 7 and 12 years and were enrolled in ordinary schools. Children with school failure, having or have had a psychiatric disorder were not included in our study.

An exhaustive sampling method was conducted in four primary schools, eight daycare centers and one cultural center distributed in five governorates of the north of Tunisia for the general population and in the department of children and adolescents' psychiatry in Razi Hospital (Tunis) for the clinical population. In total, we surveyed the parents of 206 children of the general population, and 47 children being followed for autism spectrum disorder without intellectual and language impairment.

### Material

The TESC is a hetero-questionnaire for parents. This form was adopted to alleviate the difficulties of reading and comprehension of children (16). It was initially composed of 26 items written in dialectal Arabic to improve comprehension and to better adapt to cultural and vocabulary nuances. Items were rated on a four-point Likert scale ranging from “strongly agree” to “strongly disagree.” Negative and affirmative forms were used, and all basic emotions defined by Ekman were included (17). The items were initially divided into four theoretical domains of empathy: affective, cognitive, behavioral, and somatic. TESC was developed by child psychiatrists having not <15 years of experience and was inspired from *Interpersonal Reactivity Index* (18), *Empathy Quotient* (6); *The Cognitive, Affective and Somatic Empathy Scales* (8), *Basic empathy scale* (19) *and Children's Empathy Quotient and Systemizing Quotient* (9). We adapted our version to culturally relevant situations.

### Procedure

After ethical approval of the study protocol, we obtained authorization of each institution. Parents had to read and sign an informed consent form explaining the goal of the study and confidentiality of their data. Clinical evaluation completed by the administration of categorical analysis and vocabulary B tests of EDEI-A was administered to assess children (20). This clinical evaluation's objectives were to ensure that children from general population were neurotypical and did not suffer from any psychiatric disorder, in particular neurodevelopmental disorder. For the clinical population, clinical evaluation was conducted to check that the child did not suffer from a comorbidity. We did not use Wechsler Intelligence Scale for Children (WISC) because unlike the EDEI, it's still not adapted or standardized to the Tunisian population.

### Validation Study

#### Face Validity and Content Validity

Face validity and content validity were assessed by a committee of experts after the questionnaire was written and by users during the pre-test stage (21). In our work, the expert panel was composed of seven researchers: five psychiatrists and two clinical psychologists. The panel had to study the questionnaire as a whole and then each of the items and to make changes when necessary, considering the criteria of clarity, relevance, and discrimination. TESC initially included nine items testing affective empathy, seven items for cognitive empathy, seven items for empathic behaviors and three control items testing somatic empathy. Different components of empathy were randomly distributed in the questionnaire.

#### Beta Study and pre Validation Study

A preliminary version of the questionnaire was submitted to parents of 20 neurotypical children in its paper and pencil form, and then in its computerized form to the parents of 60 neurotypical children related to our target population. The purpose of this step was to draw out the maximum number of remarks to judge the intelligibility and relevance of the different items, their acceptability to the target population and finally to adjust the scoring system (22).

#### Construct Validity

The validity of the construct was evaluated through the study of the internal structure of the questionnaire. Aprincipal axis exploratory factor analysis (EFA) with “varimax” rotation was performed to study the internal structure of the questionnaire and to extract the test major factors. Fitness of data for analysis was checked through Kaiser-Meyer-Olkin (KMO) and Bartlett's sphericity. This test is needed to check the sample size adequacy. Sampling adequacy was considered ≪ good ≫ for a KMO ≥ 0.5 (23).

#### Internal Consistency

The reliability of the TESC was assessed by Cronbach's alpha coefficient to evaluate the internal consistency and correlations between items and total score were evaluated using Pearson's r-correlation coefficient (24). Test-retest reliability was not evaluated due to COVID-19 restrictions.

#### Sensitivity and Specificity Study

The study of the sensitivity and specificity of TESC for ASD was carried out through ROC curve analysis and the measurement of the area under the curve (AUC). According to Delacour et al., the ROC curve reflects the diagnostic performance of a test. In his study, he distinguishes between tests with zero contribution AUC = 0.5, low information 0.5≤AUC <0.7, medium information 0.7≤AUC <0.9, 0.9≤ high information <1, and perfect AUC =1 (25).

### Statistical Analysis

Statistical data were analyzed using the Statistical Package for Social Sciences (SPSS) program in its 23rd version for Windows. Qualitative variables were described using means, standard deviations, and limits. Quantitative variables were described using proportions and percentages.

## Results

### Population

Of the 206 recruited children from the general population, five children diagnosed with specific learning disabilities and post-traumatic stress disorder were not included. Based on our exclusion criteria, four children from the general population and 16 of the clinical population were excluded.

Our final sample of general population was composed of 197 children with mean age of 9.11 ± 1.4 and sex ratio (M/F) = 0.6. As for clinical population, 31 children remained in the study, their mean age was 9.5 ± 1.4 and sex ratio (M/F) = 9.3.

Responding parents for both groups were mainly mothers with 65.3% in general population and 55.6% in clinical population.

### Validation Study

#### Face Validity and Content Validity

After several consultations among the experts, eight items that were deemed irrelevant and that seemed to assess other concepts besides empathy, such as education or morality, were removed. Most parents were not able to assess quantitatively or qualitatively whether their children showed the signs sought by the somatic empathy items. This led to the removal of these items. At the end of the beta study, two items that were subject to confusion and that we had to explain, one item that included a gender-determining term seemed to assess the empathic reaction to a same-sex or opposite-sex stimulus were reworded. The duration of the test was 8–10 min.

#### Construct Validity

Inter-item correlation study showed that all the selected items had a good variance. The KMO index was 0.692 and Bartlett's test of sphericity showed significance *p* < 10^−3^, we could therefore reject the null hypothesis that the correlation matrix was an identity matrix and that there was no relationship between the items. An EFA by referring to the eigenvalues >1 on the remaining 15 items was performed. This analysis allowed us to extract 4 areas explaining 43.0% of the total variance including some differences with our initial theoretical distribution: Empathic behaviors, affective empathy, cognitive empathy, and combined affective-cognitive domain (Table 1).

**Table 1:** Subdomains of the TESC as determined by factor analysis.

**Factor**	**1**	**2**	**3**	**4**
Subdomains^*^	Empathic behaviors	Affective empathy	Cognitive empathy	Combined domain
Corresponding items	6-13-16-20-23-25	1-3-10-21	2-8-9	12-18

* *Subdomains determined by factor analysis*.

### Subdomains and Total Score Inter-Correlation Matrix

Each of the 4 sub-domains was correlated positively and significantly to the total score with an r of Pearson ranging from 0.518 to 0.792 (*p* < 10^−3^) (Table 2).

**Table 2:** Subdomains and total score correlations.

**Factor**	**1**	**2**	**3**	**4**
Domain	Empathic behaviors	Affective empathy	Cognitive empathy	Combined domain
Pearson's correlation	0.792	0.619	0.518	0.535

#### Internal Consistency

Cronbach's alpha coefficient was α =0.615 which testifies to a satisfactory fidelity of TESC. Item-total correlation matrix showed all retained items were positively and significantly correlated to the total score (Pearson's r index> 0.2 and *p* < 10^−3^).

#### Sensitivity and Specificity

**Figure 1:**
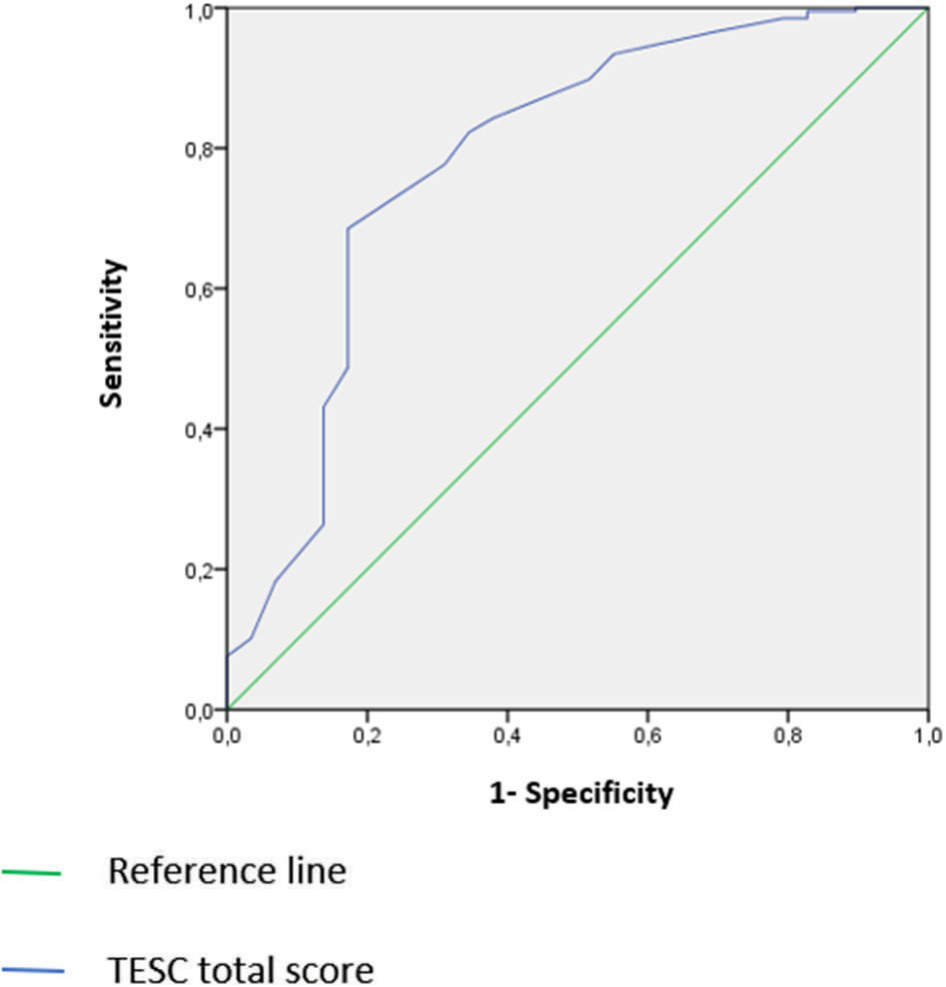
ROC curve of the total score of the TESC with clinical and general pediatric population.

The area under ROC curve was significant at AUC = 0.786 (*p* < 10^−3^), TESC is therefore moderately informative. By applying the threshold value of the total score to 16 we obtain the best sensitivity/specificity ratio: sensitivity = 84.3% and specificity = 62.1%; 95% confidence range [68.2%; 88.9%] (Figure 1).

## Discussion

After verification of the psychometric properties of the tool, TESC seems to have a satisfactory level of validity.

### Population

Our study population consisted of 197 neurotypical children and 31 children with ASD aged between 7–12 years. The KMO sample adequacy index was well above the recommended value and the sample size was sufficient for a reliable factor analysis (26). We believe that the size and age of our clinical population was comparable with that of other studies validating social cognition assessment instruments in general and clinical populations (9, 10, 27).

### Face and Content Validity

The organization of the expert committee is not clearly codified in the literature. Fermanian and Lynn emphasize the importance of having at least five experts representing the current state of knowledge, which would minimize random agreement (28, 29). The duration of the scale is considered acceptable for routine use. During this study, the experts judged the degree to which each item belonged to the concept studied as a whole and subdivided the scale into 4 domains. The initial theoretical subdivision was modified after the scale was administered to a larger sample of neurotypical children. Indeed, we realized that some items were not consistent, and we had to call upon our experts to analyze the parents' answers. This can be explained by the complexity of the concept of empathy and the blurred boundaries between its different components, which are very sensitive to the nuances of vocabulary. On the other hand, some aspects of empathy such as somatic empathy or so-called motor resonance are often measured by laboratory methods such as somato-sensory explorations, facial electromyograms, motor evoked potentials, transcranial magnetic stimulation (30).

### Construct Validity

The subdivision of the items into sub-domains determined by the EFA was not similar to our theoretical distribution. In the empathic behaviors' domain, the differences involved three items: Item 16 seems to assess the child's non-quarreling behavior when faced with a diversity of opinions. Although item 20 has a cognitive dimension, it seems to assess behaviors motivated by the cognitive reaction to the emotions of the TV character. These behaviors, which consist of the child's tendency to ask questions to his or her parents to better understand the emotional state of the character being watched. Item 23 seems to consider the child's affective reaction to a happy event happening to another person as an active affective participation and not just a simple affective sharing of another's joy.

In the affective domain, the differences involved three items as well: In item 3 and item 21, we used purely affective verbs. These items therefore explore the child's emotional state more than the empathic response they underlie. Item 10 expresses an outline of empathic behavior motivated by the emotional burden felt by the child in front of a sad person. In the cognitive domain the only difference involved item 8. The subdomains and total score inter-correlation matrix showed that the TESC assesses empathic behavior and affective empathy, better than cognitive empathy and the combined domain.

### Reliability Study

The level of reliability measured by the Cronbach's alpha index is considered acceptable for a value between 0.6 and 0.7, good for values ≥ 0.8 (31). This attests to a satisfactory level of reliability of the TESC.

### Strengths and Limits

This is the first creation and validation study of an empathy assessment tool adapted to Tunisian culture. Our general population sample size was large enough to provide interpretable results, minimizing errors due to sampling fluctuation and randomization of the sample, thus reducing selection bias. The difference between the sample size of the general population and the clinical population (197 VS 31) could be seen as a limit. However, among children with ASD (1/160) (32), children with language and without intellectual delay are a minority. This could explain the discrepancy between the two samples. More, the ratio between our two populations is comparable to those of publications dealing with validation of empathy scales in ASD (9, 33). The administration of the questionnaire directly by the study researchers and not through an online questionnaire allowed us to explain to parents and children the objectives of the study, and to avoid the exclusion of a population of parents of children who do not have access to the internet and social networks. In order to minimize sampling bias, we made sure to recruit parents of children randomly across age, gender, and location (schools, day-care, and cultural centers).

Social desirability bias has been a limitation of this work, as it can lead to over-reporting of empathic traits by parents. This limit can easily be tackled by pre-testing parents with a social desirability questionnaire. We also recommend to verify test-retst reliability, and to expand our clinical population sample by recruiting more children suffering from ASD.

## Data Availability Statement

The raw data supporting the conclusions of this article will be made available by the authors, without undue reservation.

## Ethics Statement

The studies involving human participants were reviewed and approved by Committee of Ethics of Razi Hospital, Manouba, Tunisia. Written informed consent to participate in this study was provided by the participants' legal guardian/next of kin.

## Author Contributions

HBenY administration of the test, statistical analysis, and redaction of the article. SH and AB: elaboration of the test, elaboration of the research protocol, and correction of the article. ZA and MH: elaboration of the test. MG: elaboration and administration of the test. OR, HBenM, AT, SE, and SJ: administration of the test. RF: elaboration of the research protocol and statistical analysis. AN: development of the Android application. All authors contributed to the article and approved the submitted version.

## Conflict of Interest

AN is employed by Next Gen Corp. The remaining authors declare that the research was conducted in the absence of any commercial or financial relationships that could be construed as a potential conflict of interest.

## Publisher's Note

All claims expressed in this article are solely those of the authors and do not necessarily represent those of their affiliated organizations, or those of the publisher, the editors and the reviewers. Any product that may be evaluated in this article, or claim that may be made by its manufacturer, is not guaranteed or endorsed by the publisher.
